# A Machine-Learning-Based Access Point Selection Strategy for Automated Guided Vehicles in Smart Factories

**DOI:** 10.3390/s23208588

**Published:** 2023-10-20

**Authors:** Fumiko Ohori, Hirozumi Yamaguchi, Satoko Itaya, Takeshi Matsumura

**Affiliations:** 1 National Institute of Information and Communications Technology, Yokosuka 239-0847, Japan; 2 Graduate School of Information Science & Technology, Osaka University, Suita 565-0871, Japan

**Keywords:** flexible factory, production logistics, automated guided vehicle, radio channel measurement, received signal strength indicator, link quality estimation, machine learning

## Abstract

Automated Guided Vehicles (AGVs) are becoming popular at many manufacturing facilities. To ensure mobility and flexibility, AGVs are often controlled by wireless communication, eliminating the constraints of physical cables. These AGVs require multiple Access Points (APs) to ensure uninterrupted coverage across the site. As AGVs move, they need to switch between these APs seamlessly. A primary challenge is that the communication downtime during this link-switching process must be minimal for effective AGV monitoring and control. Current AP selection strategies based on observed Received Signal Strength Indicator (RSSI) often fail in manufacturing environments due to RSSI’s inherent instability. This paper introduces a new AP selection technique for AGVs navigating these sites. Our approach harnesses the distinct movement patterns of AGVs and uses machine learning techniques to learn location-, trajectory-, and orientation-specific RSSI from the APs. Real-world factory data from our unique dataset revealed that our method extends the potential communication duration per route by 1.34 times compared to the prevalent signal strength-based switching methods commonly implemented in current drivers provided by chipset vendors or open-source Wi-Fi drivers. These results indicate that the automatic evaluation and tuning of the wireless environment using the proposed method is beneficial in reducing the time and effort required to investigate the detailed propagation paths needed to adapt AGV to existing APs.

## 1. Introduction

In manufacturing, product life cycles are accelerating. In particular, the need for low-volume, high-mix production is increasing, and it is becoming necessary to reconfigure the layout of manufacturing processes from days to months in time. In this regard, Automated Guided Vehicles (AGVs) offer significant advantages in terms of flexibility and labor savings [1]. For example, AGVs moving through diverse areas in manufacturing, logistics, warehousing, and other applications can be more flexible in layout than using conveyors that rely on fixed tracks. Furthermore, they can be adapted to transport heavy loads and objects of various shapes, which contributes to labor savings compared to using forklifts that are manually operated by human hands. In addition, they differ from humans in their ability to operate continuously, 24 h a day, 365 days a year. This minimizes downtime, the period during which production activities are suspended. AGVs have become a crucial component in the field of modern factory automation, not only for their potential to improve efficiency but also for data-driven optimization of production processes. As described above, AGVs have the potential to improve productivity, but to ensure stable operation, it is essential to control them with good-quality wireless communications.

**Figure 1:**
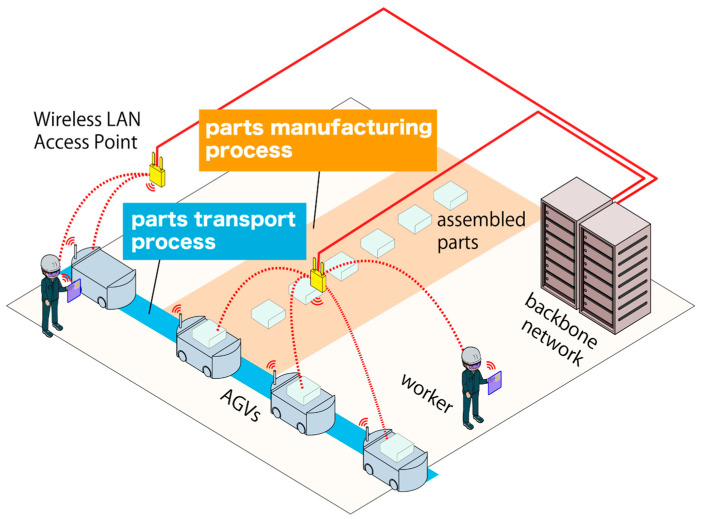
AGVs operating in a factory and their network environment.

Figure 1 shows AGVs operating in the factory environment. In this figure, AGVs are moving between the parts manufacturing process and the parts transport process, and the AGV is controlled by wireless LAN Access Points (APs). AGVs carry assembled parts brought from the parts manufacturing process to the next process through the parts transport process. By being wirelessly controlled, AGVs can eliminate the constraints imposed by physical cables, allowing for mobility and flexibility within a manufacturing or warehouse environment. Production information between the AGVs and the backbone network must be exchanged wirelessly via APs. This information includes control of the AGVs.

APs already installed in buildings are often used from a cost perspective [2]. The introduction of new APs just for AGV communication control would lead to an increase in production costs. Therefore, there is a need to control AGVs through the existing wireless LAN networks. However, if the existing APs are not suitable for AGV communication, communication difficulties will occur [3]. Efforts, including trial and error for adjustment, are necessary to ensure stable AGV communication. Once the wireless communication used for controlling safety is lost, the AGV will come to an emergency stop. In such cases, manual maintenance is required, and if trouble occurs, it will conversely increase downtime. The following describes the adjustment details in the environment shown in Figure 1.

Since each AGV needs to switch the links among those APs with their movement, Communication Loss Time (CLT), the time that AGVs are unable to communicate with APs, often appears. Self-driving AGVs use wireless communication to periodically send heartbeat signals to notify that their equipment and applications function is working. Therefore, it is desirable to minimize CLT caused by the AP switching.

In factories, there are many obstacles, such as walls and metal equipment, causing frequent switching of propagation paths between Line-of-Site (LOS) and Non-Line-of-Site (NLOS). In such situations, the Received Signal Strength Indicator (RSSI) fluctuations from surrounding APs are drastic and complex. Therefore, RSSI-based AP selection may not work in most cases. Particularly, RSSI sometimes drops quickly from peak values to below a threshold, which causes significant issues of repeated attempts to recover the connections, called “reconnection problems” [4]. Additionally, there is an observed tendency for devices to maintain a connection of non-optimal quality, even when an alternative AP in close proximity could offer a more stable and robust link. This is called “sticky problems” [5] and happens if the device cannot collect sufficient information about APs. These are the primary factors to increase CLT. Several techniques have been proposed to mitigate these problems. For example, the method in [4] models the behavior of AP switching for each environment and learns the model parameters from actual measurement data to mitigate the effects of these problems. The technique in [5] starts the switching process proactively to avoid the sticky problem. The method in [6] conducts channel scanning during non-communication time to reduce the switching time.

In the manufacturing environment, there are numerous wireless terminals in addition to AGVs. Therefore, using more scan packets like [4] may lead to further communication interference. Moreover, RSSI-based prediction models like [6,7] may not work in the manufacturing environment due to frequent LOS/NLOS status changes caused by object movements such as shutter opening and closing or metal body displacement. This may also fluctuate RSSI from a distant AP (including a sudden increase of RSSI), which compromises the algorithm. Additionally, the antenna’s directivity with AGV movement is also significant, making it difficult to judge quality based only on short-term fluctuations of RSSI.

In this paper, we propose a method to select APs for an AGV, aiming to minimize the CLT. This method predicts the selected APs in the future and aims to solve the problem of increased downtime due to AP selection errors. Furthermore, the proposed method is effective in reducing the time and effort required to adjust AGV to existing APs by automatically evaluating and adjusting the wireless environment using the proposed method.

In the proposed method, the AGV collects information on its coordinates and movement directions, as well as the signal strength from multiple APs using equipped sensing devices. The method leverages the frequently traveled route (e.g., multiple times per day) and adopts supervised machine learning to predict the optimal AP, from given labels of the optimal APs to the time-series data of RSSI from APs during past travels along the same route, the antenna directions, and the distances between the AGV and APs. By extracting features on the distance, direction, and signal strength of APs and annotating the optimal APs, the AGV can quickly choose appropriate Aps while suppressing the sticky and reconnection problems, which are expected to reduce CLT based on past experience on the route.

We have collected data from an AGV traveling along a conveyance route in a real factory and verified the effectiveness of our method. The AGV travels in a 450 m by 250 m factory building with 94 APs installed. When traveling, the AGV slows down and stops to avoid collisions with people and obstacles. Four sets of data were gathered, with three used for training purposes and the remaining one utilized for testing. The results showed that compared to a naive approach that uses only RSSI to choose APs and CLT and achieves 57.3%, the proposed one achieves 37.9%, which makes the communication time 1.34 times longer.

The contributions of this paper are two-fold.

1. It employs a data-driven, learning-based approach focusing on the movement features of AGVs in factories. Specifically, since AGVs usually follow predefined routes, the approach collects the RSSI with positions/directions of AGVs on the route and leverages their time series to learn the best APs on the routes. This is the first approach using AGV movement and RSSI from APs to choose the best APs in factory environments where the RSSI turbulence makes it challenging to make rule-based predictions.
2. The experiment has been conducted with AGV and APs data collected in an operating factory with both indoor and outdoor spaces, and the results have shown that CLT becomes 1.34 times longer than that achieved from an RSSI-based approach.

The structure of this paper is as follows: Section 2 presents related research, and Section 3 describes the proposed method. Section 4 shows the results of applying the proposed approach to the data from a working factory, and Section 5 presents an analysis of those results. Section 6 provides a summary and discusses future directions for research.

## 2. Related Work

In locations such as manufacturing sites where rapid wireless fluctuations often occur, wireless sensing technologies for collecting, analyzing, and utilizing wireless environment information are envisioned to enable mobile devices like AGVs to use wireless communication in a stable manner [8,9]. When AGVs travel while communicating with multiple wireless LAN APs in a wide indoor area, the sensing area also becomes wide. Methods for collecting wireless environment information at each point have been proposed to address this issue using many installed fixed sensors [10] or by using the same information from moving terminals [11]. In addition, system configurations and data processing procedures have been proposed to use sensing data for prediction [12]. The proposed method assumes that a sensing device is installed on the AGV and that the switching is determined on the device based on the wireless environment collected along the conveyance route. As a result, it is unnecessary to transmit wireless packets compared to issuing switch instructions via AP, which eliminates communication interference associated with switch procedures.

There are various indicators for evaluating the environment when using wireless LAN devices, but the Received Signal Strength Indicator (RSSI) is frequently used. In wireless LAN, information on the RSSI is included in the beacon packets sent by each AP at a 100 ms interval, providing a mechanism to let the terminals know the signal strength before they connect. W. Rehan et al. compiled multiple insights [13], but there are several limitations to using RSSI for estimating link quality in environments with interference [14,15,16]. Subsequent studies reported that it had a specific effect as an indicator for detecting the decrease in communication link reliability at an early stage, especially when targeting AGVs [17,18]. Furthermore, prediction methods for future RSSI trends have been verified through experiments and simulations [19,20,21,22]. In our proposed method, we also utilize RSSI as an indicator to understand wireless fluctuations. We also consider the antenna direction and the distance between the AGV and each AP to improve stability in manufacturing environments with sudden wireless fluctuations.

Like the proposed method, machine learning-based algorithms have been simulated using a dataset measured in indoor and outdoor wireless environments on mobile terminals, such as AGVs. Comparing various datasets has determined that RSSI and communication distance are more effective in characterizing the environment [23,24]. In contrast, our proposal suggests a learning algorithm to predict the appropriate AP for communication along a conveyance route from multiple APs in a manufacturing facility. This algorithm was trained using actual environmental data from a manufacturing site and verified its effectiveness. As previously mentioned, manufacturing sites present a challenging environment with frequent LOS and NLOS switches in indoor settings. In addition to RSSI and communication distance, data on the orientation of the mobile device and the AP to which it is connected is also incorporated.

To prevent the repeated occurrence of short-term connections and disconnections, this paper proposes an algorithm that makes predictions based on input data and dependencies of APs suitable for connection. Furthermore, machine learning is used as the algorithm implementation technique. This approach features the inclusion of the orientation of the AGV as input data, along with RSSI and location information between the AP and the AGV. While prior research focused on the use of RSSI and location data, we found that the inclusion of orientation information is important and contributes to prediction accuracy, especially for mobile devices that frequently change orientation, such as AGVs. For implementation, any learning technology that handles multiple, different types of time series data as input data can be used to implement. In this paper, long short-term memory (LSTM) was employed as an example of a learning technique [25]. According to existing studies, LSTM is effective in handling time-series data because it can effectively capture long-term dependencies in the data. Therefore, in this approach, LSTM was employed as a learning model-building tool because it is expected to be able to take into account the correlation of RSSI data before and after the AGV’s movement trajectory. However, the limitation of this paper is that it only uses LSTM as an example of an implementation technique and does not evaluate the properties of LSTM or the effectiveness of LSTM hyperparameters.

## 3. Integrated Approach for AP Selection

### 3.1. Metrics for AP Selection

Our method predicts an AP that AGV should use based on multiple sensing indicators. Wireless LAN terminals perform scans to detect the presence of surrounding APs and receive signal strength from them.

There are two primary methods for data collection scans: active and passive. In an active scan, the terminal initiates the process by dispatching a probe request packet. In response, the AP sends back a probe response packet carrying RSSI information. However, as the number of AGVs and/or APs increases, potential interference may arise due to the proliferation of communication packets instigated by these scans. On the other hand, during a passive scan, the terminal continually captures beacon packets broadcasted by APs to assess the signal strength. We note that given the variable intervals at which multiple APs on a single channel send out beacon packets, the scan time should be sufficient to receive the intended number of beacon packets. For instance, if an AP broadcasts beacon packets at 100 ms intervals, a logical scan time would be around 1 s to capture ten beacon packets from the AP. However, it is crucial to account for the fact that certain packets might be undetected due to wireless environment challenges.

In many manufacturing sites, both terminals and AGVs operate on the same channel, making it necessary to employ passive scans to avoid congestion. In terms of data collection, it is challenging to obtain data from multiple APs across multiple 5 GHz band channels (from 36 to 64 and from 100 to 140) using a single wireless interface. This is because scanning all channels can be time-consuming. Furthermore, since an interface can only scan one channel at a time, sudden RSSI fluctuations in other channels may be overlooked. In this paper, we employ a wireless environment sensor that can scan 20 channels simultaneously, thereby reducing the time required to scan each channel individually. This approach minimizes the time during which wireless fluctuations cannot be monitored and allows for the collection of RSSI information from beacon packets.

**Figure 2:**
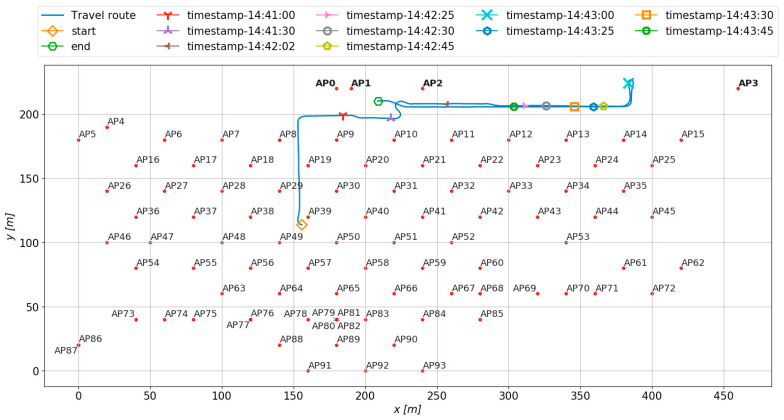
Travel route of AGV and coordinates of APs.

Figure 2 shows the environment of a target factory with 94 APs and AGVs running on the travel path around the APs. AGVs travel the route from the start point to the goal point via a partially different path for the purpose of conveying parts.

The building has an external wall located at *y* = 200, which is equipped with shutters that allow trucks and AGVs to pass through for frequent material transportation. The APs, named AP0 to AP3, are installed in a separate building located above *y* = 200. In addition, the AGV travels within the lower building and the upper roofed semi-indoor area, with *y* = 200 as the boundary. There are locations where more than one AP is installed on a single pillar, such as AP76, AP77, AP79, AP80, AP81, AP82, AP86 and AP87. In the environment, the signal strength of each AP is acquired by the developed sensor and used as an indicator for estimation. The signal strength of each AP is determined by identifying the RSSI of each AP from the beacon packets and then extracting the average signal strength per second for each AP. The AP No. of the connected AGV is obtained by the sensor.

To make use of the repeated movement of AGVs throughout the day, their 2D coordinates, *x* and *y*, are utilized as indicators. In addition, the distance between each pair of AGVs and APs is calculated using their respective coordinates, which serves as an additional indicator. We note that such an AGV to transport goods lacks a suitable location for an antenna on its upper surface. In this case, the communication antenna is installed on the side of the vehicle. However, the metal body of the AGV between the communication device and AP can cause the LOS and NLOS states to switch depending on the orientation of the AGV relative to the connected AP. This orientation also serves as an indicator since it may affect the signal strength.

### 3.2. Estimation Algorithm

**Figure 3:**
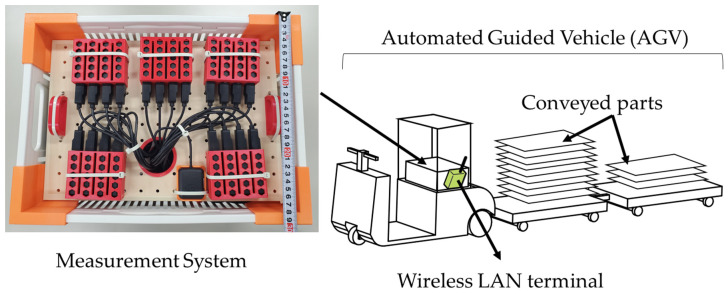
Measurement System mounted on AGV.

The factory where the experiment was conducted uses Cisco Aironet 2700 and 2800 series APs [26]. The AGV is equipped with the SX-PCEAN2 wireless module [27] for data communication, which operates with a Qualcomm Atheros AR9592 chipset. Our measurement system in Figure 3 has the ability to capture communication frames for 20 channel bands at a time. There are a total of 19 channels in the 5 GHz band of the wireless LAN: 36, 40, 44 and 48 for W52; 52, 56, 60, 64, 100, 104, 108, 112, 116, 120, 124, 128, 132, 136 and 140 for W56. Therefore, the performance of the measurement system is sufficient to collect information on all channels used in the 5 GHz band in the target environment.

**Figure 4:**
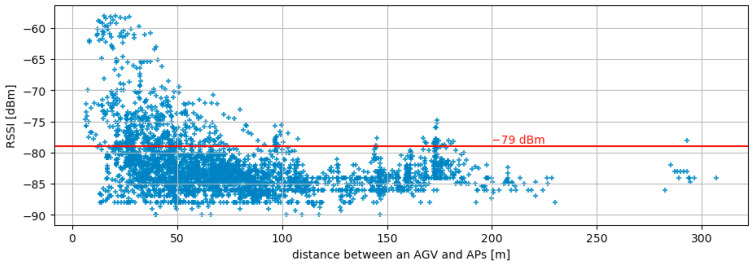
RSSI captured from measurement system vs. distance between an AGV and APs.

Target frames are 802.11 a/b/g/n type frames, which are saved in pcap format. It consists of five single-board computers; one of them is a master terminal, and the four are slave terminals. Each terminal has five wireless interfaces. The master terminal captures frames and collects captured frames from slave terminals. It is equipped with Raspberry Pi 4 as a single-board computer and the WI-U2-300D of Buffalo Corporation as a wireless interface. The measurement system targets all packets sent by the AP at 100 ms intervals for capture. From all packets, the measurement system classifies each packet according to the AP’s MAC address. The RSSI of the beacon, when it is received by the sensor, is used as input data for learning. Although the measurement system targets all packets transmitted by the APs at 100 ms intervals for capturing, the one-second average of the RSSI is used as input to the learning model described in Section 3.3. For location information, the LiDAR sensor on the AGV was used; the LiDAR data includes not only location information but also the orientation of the AGV. Furthermore, the distance between the AGV and the AP is calculated from the position obtained from LiDAR and the AP placement information shown in Figure 2. The relationship between the distance of each AP and the RSSI of packets arriving from the AP is shown in Figure 4. In the target environment, there are several locations where packets can be received above the threshold of −79 dBm, which can be considered selectable even when more than 250 m away from the AP, indicating that this is not a simple distance attenuation environment.

**Figure 5:**
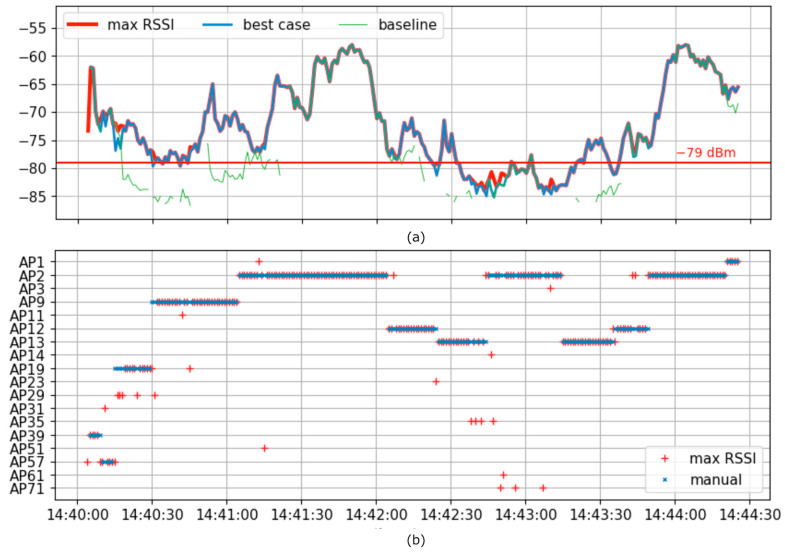
(**a**) RSSI of APs along the timeline (chosen by three algorithms). (**b**) Selected APs.

By default, this wireless module conducts active scans described in Section 3.1, and when the communication quality becomes low, it disconnects the link with the currently connected AP and searches for a new AP. This naive implementation is called “baseline”. The RSSI behavior of the baseline during the AGV traveling in the operating factory is shown in Figure 5a.

In “baseline”, CLT accounted for 150 s out of the total 262 s during the travel, representing 57.3% of the time. This was because RSSI fell below the acceptable threshold, such as −79 dB, in the context of IEEE802.11n standards. For a better understanding of the CLT, we further divided it into two sub-categories: Down Time (DT) and Repair Time (RT). During DT, the terminal remains inactive, and no efforts are made to re-establish the connection. Meanwhile, RT denotes the span wherein efforts are being made to reestablish communication with an alternate AP; hence the time is referred to as “Repair Time”. In our “baseline” scenario, the durations for DT and RT were recorded as 61 s and 89 s, respectively.

To minimize CLT, it is necessary to select the appropriate AP based on the sensing indicators. The proposed approach uses predictive techniques for the selection. Specifically, the following indicators are considered for prediction:

- Coordinates ($x_{a}$, $y_{a}$) and orientation [rad] of AGV;
- Currently connected AP *i;*
- Signal strength [dBm] of each connectable AP *j;*
- Distance [m] and orientation ($x_{k}$, $y_{k}$) to each AP *k* from AGV.

“max RSSI” in Figure 5a is a simple policy to choose the AP with the highest RSSI at each point. The RT of “max RSSI” was 58 s, which was shorter than 89 s of “baseline”. However, by examining the AP selection status shown in Figure 5b, we found that APs were switched 51 times, and in the worst case, five switching events happened during 10 s, meaning frequent disconnections. Consequently, it increases CLT, which causes the reconnection problem. Assuming that the AP switching time is 2 s for each AP, the DT is 102 s, resulting in an increased CLT of 160 s.

Ideally, the number of AP switching events should be minimized while the feasible RSSI is maintained, like the “best case” in Figure 5. The best case is deduced from historical data to ensure future RSSI quality surpasses the −79 dBm threshold. Specifically, the AGV selects the AP distinguished by the highest and most prolonged sustained RSSI quality, minimizing the frequency of AP selections throughout the entire conveyance route. This AP selection strategy can be extrapolated from the historical data collected during the AGV’s route from start to end. Therefore, we propose a predictive approach wherein we forecast the most suitable AP for the future based on the APs that have demonstrated superior performance in the past, along with current information. This forecasting is contingent upon a correlation between historical and current AGV travel trajectories and variations in the wireless environment. However, in implementing this algorithm, it is necessary to understand the past RSSI variations along the trajectory of the AGV, integrate multiple data based on location information, predict where the AGV will move next, make decisions and adjust threshold values based on consideration of trade-offs among multiple data. Therefore, to realize the proposed algorithm, we decided to use machine learning as a technique to derive the optimal one from the correlation of multiple input data.

Our approach automates extracting several indicators from some changes and integrates them to bring the prediction results closer to the best AP that AGV should use. The indicators are the coordinates and orientation of the AGV, the currently connected AP, the signal strength of each connectable AP, and the distance and orientation of each AP from the AGV. To automate this process on the AGV and mimic the best behavior, we propose a machine learning-based approach that uses manually annotated data experienced on routes in the past. Again, the idea is to learn the patterns of RSSI that are reproduced when traversing the same route. It predicts the AP that should be selected after *S* seconds, which should be longer than the AP switching time.

### 3.3. Machine Learning Model Architecture

**Figure 6:**
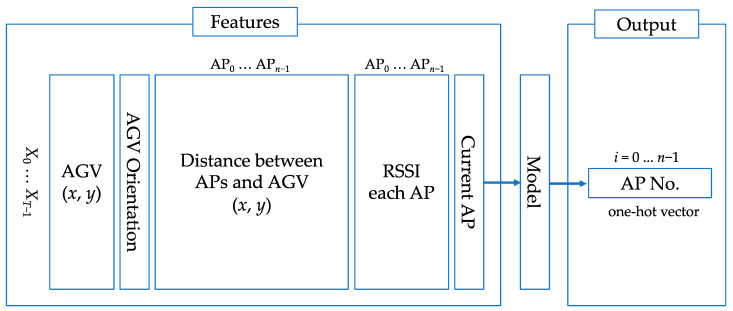
Model structure.

The learning process is performed using the sensing indicator vectors presented in Section 3.2. For each prediction, the time series of each index of the last *T* seconds is given to the learning model, which identifies the AP to select *S* seconds later. The input/output data structure is shown in Figure 6. Since the coordinates, distance, and RSSI values depend on the environment, we normalize them to a range of −1 to 1 based on their maximum and minimum values. Before normalization, the coordinates x range from a maximum value of 500 m to a minimum value of 0 m. The coordinates y range from a maximum value of 240 m to a minimum value of 0 m. The RSSI values range from a maximum value of −50 dBm to a minimum value of −95 dBm. The orientations of AGV are in the range of π to −π. To represent missing RSSI values due to packet loss or other reasons, we use −30 so that we can distinguish it from the normal values. We evaluate the performance for each of 262 −S samples. In addition, for the predictions after the S-th evaluation, we use the predicted AP as the current AP. To prevent overfitting, we stop training when the number of epochs exceeds 60 and there is no improvement in the value.

The proposed method uses the orientation of the AGV as input data for the following two reasons. Firstly, depending on the placement of the wireless LAN terminal on the AGV, there is a risk that the propagation path will be in NLOS due to the presence of the AGV’s body between the AP and the wireless LAN terminal. In such instances, the RSSI may change and become more dependent on the change in orientation than on the coordinates of the AGV, in which case the prediction accuracy will be degraded. Therefore, the proposed method identifies the correlation between the orientation of the AGV and RSSI variations by incorporating orientation information as input data, which improves prediction accuracy. Secondarily, different values of orientation can help predict the next movement of an AGV.

The values of each indicator mentioned in Section 3.2 and the annotated values described in Section 3.3 of the last *T* seconds are given for the model training. Denoting the number of APs as *n*, the input data has T×(3n+4) elements. The output of the prediction is the AP to select after *S* seconds, represented in the form of a one-hot vector of length *n*. Regarding the datasets presented in Table 1, No. 1 was used as test data; 90% of the samples from No. 2 to No. 4 were used for training and the remaining 10% for validation. All datasets were obtained on the travel path, and the number of available APs was 94. It is important to note that our study is tailored to a real-world manufacturing environment, where collecting a substantial number of samples is very difficult and time-consuming. It should also be taken into account that data collection in an operating plant is constrained, and the available data is limited. A skewed distribution in the data set could bias the training of the model. Therefore, a data ratio of 90% for training and 10% for validation was chosen in order to train more data and obtain more balanced results, even with limited input data.

**Figure 7:**
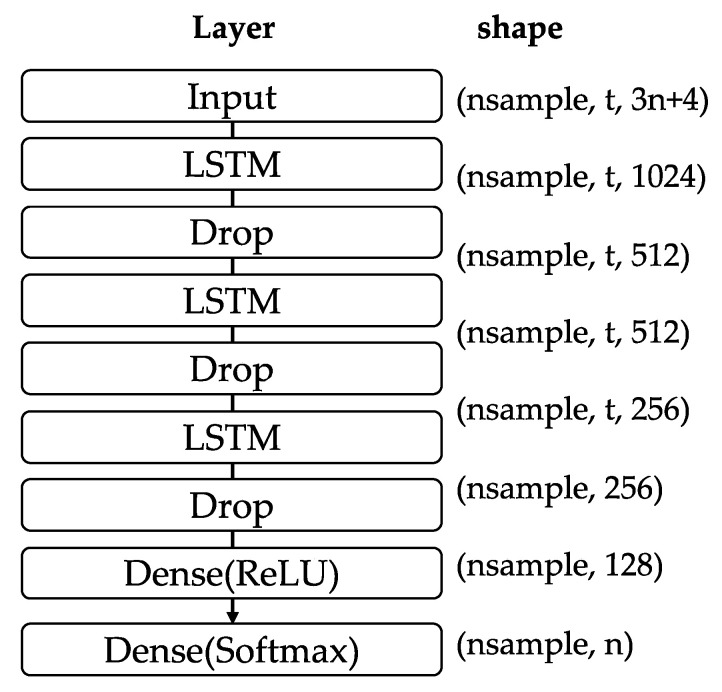
Layers of study model.

The learning model employs three layers of LSTM (shown in Figure 7) with multiple drop layers to prevent overfitting. A Softmax layer is inserted to ensure that the sum of the outputs is 1 and the value of each element in the one-hot vector lies between 0 and 1. The model output has the *n* classes, and the loss function categorical_crossentropy is used to indicate the most suitable AP with the maximum probability. Other hyperparameters are presented in Table 2. The algorithm was implemented using scikit-learn, a Python library [28].

## 4. Experiment and Results

We calculate the precision, recall, accuracy, and F1-score values for evaluation. The proposed method was trained and validated on datasets No. 2 and No. 3, achieving a precision of 0.80, recall of 0.80, F1-score of 0.75, and accuracy of 0.80.

**Figure 8:**
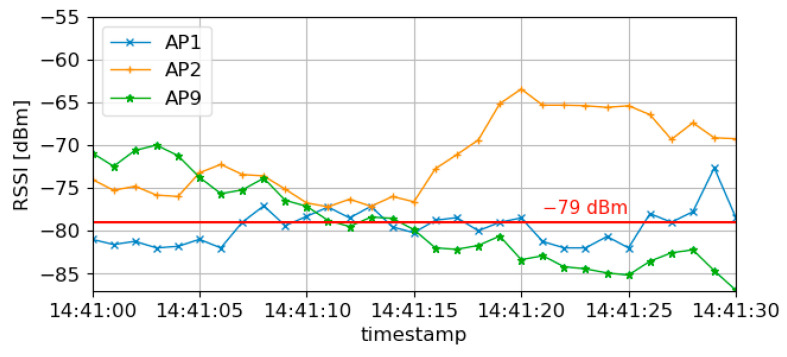
RSSI changes of AP1, AP2, and AP9.

The prediction results of the three prediction methods with different input data compared to best case are shown in Figure 8 and Table 3.

It shows (1) the best case and the results of three prediction methods: (2) the method that only uses RSSI for prediction [12], (3) the method that excludes the orientation of the AGV for prediction, and (4) the proposed method.

Method (4) indicated switching from AP9 to AP2 after 14:41:15. Method (2) suggested changing from AP9 to AP1 at 14:41:15 and then to AP2 at 14:41:20. However, method (3) did not indicate changing at the time, 14:41:15 and then to AP2 at 14:41:20. As a result, in method (2), reconnection to three different APs was experienced during 5 s, while method (3) resulted in sticking to a poor-quality AP. The proposed method (4) avoided these problems, and in particular, it avoided the short-term reconnection problem by choosing AP2 instead of AP1 at 14:41:15, when method (2) chose AP1 at the same time.

**Figure 9:**
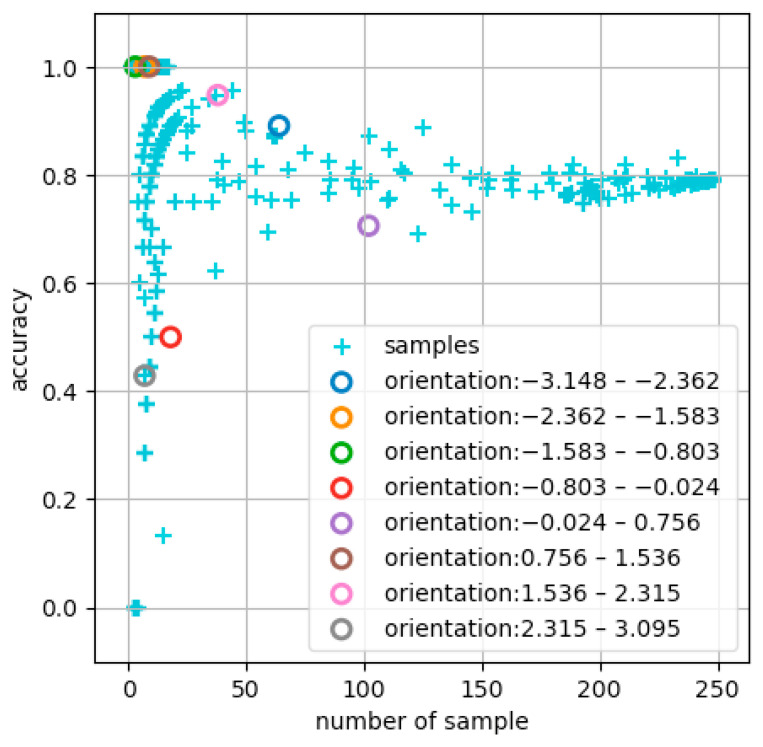
Number of data samples vs. prediction accuracy.

The proposed method (4), including orientation, is closest to the best case in (1). The impact of different input data on the AP prediction accuracy is shown in Figure 9.

Figure 9 shows the analysis of estimation accuracy for the input data. The features in each column of Figure 6 are divided into eight bins by value. For each bin, the number of samples and average estimation accuracy are determined, and the relationship is shown in Figure 9. As the number of samples increases, the estimation accuracy converges around 0.8, but as the number of samples decreases, the estimation accuracy decreases. AGV orientation is a value in radians; 8 divisions mean the bin is divided into 8 orientations. When moving in the lower-right orientation of Figure 2 with an orientation angle between −46 to 1.37 degrees (−0.803 to −0.024 rad) and in the upper-left orientation with an angle between 132 to 177 degrees (2.315 rad to 3.095 rad), the number of samples is less than 25, and the accuracy is below 0.6. For example, when the AGV is moving in the lower right orientation around 14:42:20 in Figure 2, both the number of samples and accuracy are low. This finding suggests that incorporating techniques for imbalanced data may significantly improve the success rate when the orientations for AGV travel routes are biased.

Table 4 shows (1) the best case and the CLT results of three prediction methods: (2) the method that only uses RSSI for prediction [12], (3) the method that excludes the orientation of the AGV for prediction, and (4) the proposed method.

Table 4 shows (1) the best case and the CLT results of three prediction methods: (2) the method that only uses RSSI for prediction [12], (3) the method that excludes the orientation of the AGV for prediction, and (4) the proposed method. The RTs of all the methods were at maximal 80 s, which were shorter than 89 s of the baseline shown in Figure 5. The DT was calculated based on the time required for two types of reconnections: when the RSSI could not receive the AP signal for 1 s and when the AP was switched due to prediction. The number of reconnections was 17, 22, and 7 for methods (2), (3), and (4), respectively. Assuming that reconnection time was 2 s, the DT was 14, 34, and 44, respectively. As a result, the CLT was in the order of (4) < (2) < (3), and the proposed method achieved the smallest CLT value. In method (4), the LSTM learning model was trained with *n* = 94, *T* = 10 and *S* = 5 to select APs. The prediction period was 262 − 10 − 5 + 1 = 248 s. In method (4), the CLT ratio was 37.9% (94 over 248 s), leading to an improvement of 4.8% compared to that by method (2). Furthermore, with the “baseline” scenario shown in Section 3.2, the CLT ratio was 57.3% (150 over 262 s). Therefore, the CLT ratio of the proposed method is improved by 19.4%, which leads to 1.34 times longer communication time than that implemented on actual devices.

## 5. Analysis and Discussion

In this paper, real data from approximately 4.5 min of AGV movement was used to evaluate the proposed method. The scenario in (1), the best case for the entire route, would result in 11 times AP selections and a 31% CLT rate, and our proposed approach is the closest to these values. The input data have a time resolution of one second and spatial resolution of the order of meters. These resolution requirements are consistent with existing research [12] that focuses on wireless quality metrics with a time resolution of one to 10 s and a spatial resolution of one meter. As a result, our method is considered to be feasible. Furthermore, the approach can be made more versatile by extending the data handling in time and space, although this may entail limitations in some environments. The five limitations associated with the use of this proposed method are described below:

The first limitation is that rapid RSSI fluctuations of less than one second cannot be used for learning. In this study, the results outlined in Section 4 were achieved through the learning of information at a time resolution of approximately one second. Although the results averaged from data within one second were used as input, if forecasts are to be made in an environment where large RSSI fluctuations occur within one second, a method to utilize beacon information transmitted from APs at 100 ms intervals without averaging in one-second cycles should be considered. However, not all beacon information can be received in all time spans, so it is necessary to take countermeasures against anomalous values that occur in sections where they cannot be received.

The second limitation is that it is difficult to learn when there is less data in a certain direction; if the estimation accuracy for local movements of AGVs can be increased by improving the amount of data or the method, it will be possible to respond to various movements of AGVs along the entire route. Although outside the scope of this paper, AGVs that move in the *z*-direction as well as in the *xy*-direction will affect the installation height of the AP. In this case, the scope of this proposal should be extended to the z-direction.

The third limitation is the potential for pre-training selection errors in environments where similar routes include unpredictable RSSI variations that are not reproducible and have low correlation. For example, this occurs in scenarios where the direction of the AGV suddenly changes, or the RSSI fluctuates in a way that was not present in the historical data. In the absence of correlation, AP selection may not perform optimally. Even if a selection error occurs in the most recent environment, if there is a correlation in subsequent runs, the model can be re-trained with the latest data to bring it closer to the optimal value.

The fourth limitation is the possibility of achieving the best scenario by fixing the AP selection at each location without using the proposed method. This relates to a scenario in which RSSI variations and AGV movements are completely computer-controlled, and the AGV travels along fixed rails. In such cases, the best case can be achieved because the best AP selection is determined for each location, orientation, and time. However, fully controlled environments are rare. In the case of AGVs with autonomous routes, there will be discrepancies in the passing and waiting times at each point. Therefore, it is believed that there are few real-world environments that fall under this constraint.

Finally, the fifth limitation is that a comprehensive comparative evaluation of hyperparameters, such as dropout rates, introduces LSTM forgetting mechanisms, and the findings from them are not available in this paper. However, it is worth noting that the model employed in this study is built on a basic type with three LSTM layers. This configuration is considered to be the basic configuration for using neural networks for training. As a result, similar results could be obtained using other models, such as 2D-CNN and DNN.

## 6. Conclusions

For AGVs to travel over a wide range of a manufacturing sites, they need to seamlessly select APs. A challenge is that the communication downtime during this link-switching process must be minimal for effective AGV monitoring and control. In this paper, a new AP selection technique for AGVs moving was presented to address this challenge. In this technique, three prediction methods were compared using CLT as an indicator: (2) predicated solely on RSSI, (3) prediction based on (2) plus location information, and (4) proposed prediction based on (3) plus orientation information. The CLT downtime caused by AP selection errors was in the order of (4) < (2) < (3), and the proposed method (4) is closest to the optimal scenario and can effectively minimize CLT. The CLT ratio for the whole travel time was 37.9% for the proposed method (4), an improvement of 4.8 points compared to the method (2). The CLT ratio improved by 19.4 points compared to 57.3% for the conventional method shown in Section 3.2, and the communication time increased by 1.34-fold. As a result, based on the evaluation results using realistic factory measurement data, we can conclude that the proposed method is effective for optimal AP selection with the goal of minimizing the CLT because it can predict future AP selection and reduce downtime due to AP selection errors. Because the proposed method was effective without investigating the detailed propagation paths required to adapt AGV to an environment with multiple existing APs, it has the potential to reduce the time and effort required to automatically evaluate and adjust the wireless environment for AGVs in the future.

## Figures and Tables

**Table 1:** Summary of the data set.

No	Number of Samples	DT [s]	RT [s]	Details
1	262	22	59	Test: 100%
2	331	26	59	Train: 90%, Validation: 10%
3	555	27	72	Train: 90%, Validation: 10%
4	632	32	58	Train: 90%, Validation: 10%

**Table 2:** Hyperparameters.

Hyperparameters	Values
Loss function	categorical_crossentropy
Optimization function	Adam (lr = 0.0001)
Dropout Rate	0.6
Number of Batches	64
Number of Epochs	<300
Number of APs: *n*	94
Time to input: *T* [s]	10
Time to predict: *S* [s]	5–95

**Table 3:** AP prediction of three prediction methods.

Time	14:41:10	14:41:15	14:41:20	14:41:25	14:41:30
(1) best case	AP9	AP2	AP2	AP2	AP2
(2) prediction based solely on RSSI	AP9	AP1	AP2	AP2	AP2
(3) based on (2) plus location information	AP9	AP9	AP2	AP2	AP2
(4) based on (3) plus orientation information	AP9	AP2	AP2	AP2	AP2

**Table 4:** Evaluation results.

Algorithm	F1-Score	RT [s]	DT [s]	CLT [s]	CLT Rate [%]
(1) best case	-	59	22	81	31.0
(2) prediction based solely on RSSI	0.90	72	34	106	42.7
(3) based on (2) plus location information	0.92	65	44	109	44.0
(4) based on (3) plus orientation information	0.75	80	14	94	37.9

## Data Availability

The datasets generated and/or analyzed during the current study that have been approved for publication by the factory are available from the corresponding author on reasonable request.
